# Triptolide Attenuates Podocyte Injury by Regulating Expression of miRNA-344b-3p and miRNA-30b-3p in Rats with Adriamycin-Induced Nephropathy

**DOI:** 10.1155/2015/107814

**Published:** 2015-05-20

**Authors:** Chun-Bo Jiang, Ming-Gang Wei, Yue Tu, Hao Zhu, Chun-Qing Li, Wei-Min Jing, Wei Sun

**Affiliations:** ^1^Department of Graduate School, Nanjing University of Chinese Medicine, Nanjing 210023, China; ^2^Department of Nephrology, Suzhou Hospital of Chinese Medicine, Suzhou 215000, China; ^3^The First Affiliated Hospital of Soochow University, Suzhou 21500, China; ^4^Department of Nephrology, Wuxi No. 3 People's Hospital, Wuxi 214000, China; ^5^Department of Nephrology, Jiangsu Provincial Hospital of Chinese Medicine, Affiliated Hospital of Nanjing University of Chinese Medicine, Nanjing 210029, China

## Abstract

*Objectives*. We investigated the action of triptolide in rats with adriamycin-induced nephropathy and evaluated the possible mechanisms underlying its protective effect against podocyte injury. *Methods*. In total, 30 healthy male Sprague-Dawley rats were randomized into three groups (normal group, model group, and triptolide group). On days 7, 28, 42, and 56, 24 h urine samples were collected. All rats were sacrificed on day 56, and their blood and renal tissues were collected for determination of biochemical and molecular biological parameters. Expression of miRNAs in the renal cortex was analyzed by a biochip assay and RT-PCR was used to confirm observed differences in miRNA levels. *Results*. Triptolide decreased proteinuria, improved renal function without apparent adverse effects on the liver, and alleviated renal pathological lesions. Triptolide also elevated the nephrin protein level. Furthermore, levels of miR-344b-3p and miR-30b-3p were elevated in rats with adriamycin-induced nephropathy, while triptolide treatment reversed the increase in the expression of these two miRNAs. *Conclusions*. These results suggest that triptolide may attenuate podocyte injury in rats with adriamycin-induced nephropathy by regulating expression of miRNA-344b-3p and miRNA-30b-3p.

## 1. Introduction

Recent estimates indicated that the number of patients with chronic kidney disease (CKD) in China is about 119 million [1], which causes a great burden to the society and economy. Glomerular disease continues to be the leading cause of CKD in China [1]. Recently, the damage and repair of podocytes has become a hot topic in kidney disease research. It has been shown that podocytes are an important part of the glomerular filtration membrane and their injury is not repairable. It is known also that damage to podocytes plays a vital role in the development of glomerular sclerosis [2, 3].

Nephrin is an important protein located in the glomerular filtration barrier. It was discovered by the research team led by Tryggvason [4] in 1999, and, now, it is well known that nephrin is the most important transmembrane glycoprotein in the podocyte slit aperture membrane [5]. Nephrin is a protein molecular marker of glomerular podocytes [6] that belongs to the immunoglobulin cell adhesion molecules superfamily and contains 1241 amino acid residues, comprising an extracellular domain, a transmembrane region, and a carboxy-terminal cytoplasmic tail. Downregulation of nephrin expression alters the slit membrane structure, causes podocyte injury, and produces a substantial amount of proteinuria, leading to the development of glomerular sclerosis and renal function damage.

Triptolide is a product of epoxidation of two terpene lactone compounds extracted from the traditional Chinese herb* Tripterygium wilfordii*. It has been demonstrated that triptolide has immunomodulatory and anti-inflammatory effects [7, 8]. Animal studies show that triptolide can reduce proteinuria and effectively protect podocytes in rats with puromycin-induced nephropathy [9]. The protective effect may be associated with upregulation and distribution of nephrin and podocin [9]. Our preliminary study showed that* Tripterygium* preparations could ameliorate proteinuria due to enhanced expression of nephrin [10]. Recently, some studies suggested that triptolide might exert its pharmacological effect via modulation of microRNAs (miRNAs). It was reported that triptolide may regulate heat shock protein 70 by inducing the expression of miR-142-3p [11]. Another study revealed that triptolide induces apoptosis in T-cell lymphocytic leukemia cells probably by downregulating miRNA-16-1 [12].

miRNAs are a set of endogenous small molecules, 20–22 nucleotides in length, which are widely present in plants, animals, viruses, and humans. It is well established that miRNAs participate in a multitude of developmental and cellular processes in worms, flies, and plants, by either repressing translation or causing miRNA degradation [13–16]. Furthermore, miRNAs play an important role in physiological and pathological processes such as cell metabolism, cell signaling, and others [17, 18]. Previous studies have shown that miRNAs can modify gene expression [19], and it has been found that miRNA deregulation is correlated with major kidney diseases [20]. For example, in rats with ischemic acute kidney injury (AKI), miR-21 was induced after renal ischemia in kidney tissues, and miR-494 was upregulated [21, 22]. Although previous studies showed the importance of miRNAs in the regulation of kidney physiological functions and kidney homeostasis [23], the exact identity of such miRNAs remains largely unknown.

In this study, we utilized a rat model of adriamycin-induced nephropathy and examined effects of pharmacological intervention with triptolide. We elucidated possible protective mechanisms of triptolide by means of the biochip technology. We found that triptolide attenuated podocyte damage by regulating expression of miRNAs.

## 2. Materials and Methods

### 2.1. Animals

In total, 30 healthy 6–8-week-old male Sprague-Dawley (SD) rats weighing 180–200 g were provided by the Shanghai Silaike Experimental Animal Center (Shanghai, China, License SCXK (Hu) 2013-0003). All animals were housed at 22 ± 3°C and 50% ± 10% humidity and were given free access to tap water and standard chow.

### 2.2. Reagents and Instruments

Triptolide (>99% purity) was purchased from the National Institute for the Control of Pharmaceutical and Biological Products and dissolved in dimethyl sulfoxide. Adriamycin was purchased from Zhejiang Hisun Pharmaceutical Co., Ltd. (Zhejiang, China). The microalbuminuria detection kit was purchased from Shanghai Sunred Biological Technology Co., Ltd. (Shanghai, China). Primary and secondary antibodies against nephrin were obtained from Santa Cruz Biotech (Santa Cruz, CA, USA). RT-PCR kits were purchased from RiboBio (Guangzhou, China).

### 2.3. Animal Model and Experimental Design

After 1 week of adaptive feeding 30 rats were randomized into three groups (*n* = 10 for each group) as follows: normal group, model group (adriamycin-induced nephrotic rat model group), and model + triptolide treatment group (triptolide, 200 *μ*g/(kg × d)). Rats in the model and treatment groups were intravenously injected with 0.2% adriamycin at a dose of 6 mg/kg [24]. After 1 week, 24 h urine samples were obtained in metabolic cages to measure, urinary protein excretion and the model was considered to be successfully established when proteinuria >50 mg/kg per day. Subsequently, rats in the model and normal groups were daily administered normal saline (NS) solution at a dose of 1 mL/100 g for 8 weeks, whereas rats in the triptolide group were daily administered 200 *μ*g/(kg × d) triptolide for 8 weeks. 24 h urine samples were obtained on days 7, 28, 42, and 56 in metabolic cages; and all rats were sacrificed on day 56.

### 2.4. Testing Index and Methods

#### 2.4.1. Kinetics of Urinary Protein Excretion

On days 7, 28, 42, and 56, after establishment of the adriamycin-induced nephropathy model, 24 h urine samples were collected and urine protein concentrations were determined by immunoturbidimetry.

#### 2.4.2. Serum Biochemical Parameters

On day 56 after establishment of the adriamycin-induced nephropathy model, all rats were anesthetized with ketamine and blood samples were collected from their hearts by cardiac puncture. Sera were separated for the detection of serum albumin (Alb), serum creatinine (Scr), blood urea nitrogen (BUN), and alanine aminotransferase (ALT) by a HITACHI7080 automatic biochemical analyzer.

#### 2.4.3. Light Microscopy Examination

Renal cortices were separated, fixed in 10% phosphate buffered formalin solution, embedded in paraffin, cut into 2-*μ*m thick slices, and stained with a periodic hematoxylin and eosin reagent. Pathological changes of the kidney were observed under the light microscope.

#### 2.4.4. Electron Microscopy Examination

Renal tissue samples were dehydrated. The renal cortexes were cut into 1 mm × 1 mm × 1 mm portions, fixed in 3.75% glutaraldehyde, washed in PB and post-fixed in 1% osmic acid, embedded in epoxy resin, stained with uranyl acetate and then with citric acid, and examined and photographed using a HITACHI7500 transmission electron microscope (Olympus, Tokyo, Japan).

#### 2.4.5. Immunohistochemistry

The immunohistochemical ABC method was used. PBS solution, instead of the primary antibody, was used as the negative control. Semiquantitative analysis of nephrin was performed using the MOTIC pathological image analysis system. Ten random fields of each specimen were selected and digitized for analysis. Then, the optical density of nephrin immunohistochemical staining was calculated and the positive integral optical density was identified. Higher positive integral optical densities corresponded to stronger levels of protein expression. The percentage of positive stained area occupying the selected glomeruli was calculated. Aggregated data were analyzed using SPSS 14.0 software.

#### 2.4.6. Western Blot Analysis

Renal tissues were homogenized and disrupted, and the supernatant was isolated. The protein samples were put into SDS-PAGE loading buffer and then transferred to a PVDF membrane after separation. Proteins were blocked with 10% skim milk for 2 h and then incubated with rabbit anti-rat nephrin polyclonal antibodies overnight at 4°C. The proteins were allowed to react with the corresponding horseradish peroxidase- (HRP-) labeled goat anti-rabbit IgG (diluted with TBST to 1 : 5000) for 1 h at room temperature. After extensive washing, proteins were mixed with the ECL reagent, and the experimental results were recorded by X sheets. Beta-actin was used as an internal reference, while nephrin band intensity was calculated from gray values of computer images processed by the image analysis software, which indicated the extent of nephrin protein signal.

#### 2.4.7. Gene Chip Technology Analysis

Total RNA was isolated using TRIzol (Invitrogen) and purified with an RNeasy mini kit (QIAGEN), according to manufacturer's instructions. RNA quality and quantity were evaluated using a NanoDrop spectrophotometer (ND-1000, NanoDrop Technologies), and RNA integrity was determined by gel electrophoresis. RNA labeling and array hybridization was carried out according to Exiqon manual. After quality control, the miRCURY Hy3/Hy5 Power labeling kit (Exiqon, Vedbaek, Denmark) was used according to the manufacturer's guidelines for miRNA labeling. After termination of the labeling procedure, Hy3-labeled samples were hybridized on the miRCURYTM LNA Array (v. 18.0) (Exiqon) according to the array manual. Then the slides were scanned using an Axon GenePix 4000B microarray scanner (Axon Instruments, Foster City, CA). Scanned images were imported into GenePix Pro 6.0 software (Axon) for grid alignment and data extraction. Replicated miRNAs were averaged and miRNAs with intensities ⩾30 in all samples were chosen for calculating the normalization factor. Expressed data were normalized using median normalization. After normalization, miRNAs that were differentially expressed between the two groups were identified by significant fold change and *P* values. Finally, hierarchical clustering was performed to show distinct miRNA expression profiles among samples. (This work was done by Shanghai Kangcheng Biological Technology Co., Ltd.)

#### 2.4.8. miRNA Validation by Reverse-Transcription PCR

Total miRNA was extracted from rat kidney and detected according to the TRIzol kit instructions. Reverse transcription PCR (RT-PCR) was performed by using an RT-PCR kit, according to the manufacturer's protocol. The sequences of miRNA-30b-3p and miRNA-344b-3p were obtained from RiboBio. First, a special RT-primer for miRNAs was used for reverse transcription at 42°C for 1 h and at 72°C for 10 min. Then the miRNA PCR sequence comprised 94°C for 3 min, 94°C for 30 s, 60°C for 20 s, 72°C for 30 s, and 72°C for 10 min. The number of cycles was 35. U6 snRNA was used as an internal reference. PCR products were extracted and identified by 1.2% agarose gel electrophoresis, and the results were observed using a gum electrophoresis instrument.

#### 2.4.9. Statistical Analysis

Statistical analyses were performed using SPSS14.0 software. All data are presented as the mean ± standard deviation (SD). Statistical significance among groups was determined by one-way analysis of variance (ANOVA). Comparisons between groups were made using standard Student's *t*-test. Differences of *P* < 0.05 were regarded as significant.

## 3. Results

### 3.1. Triptolide Reduced Urinary Protein Excretion in Rats with Adriamycin-Induced Nephropathy

**Figure 1:**
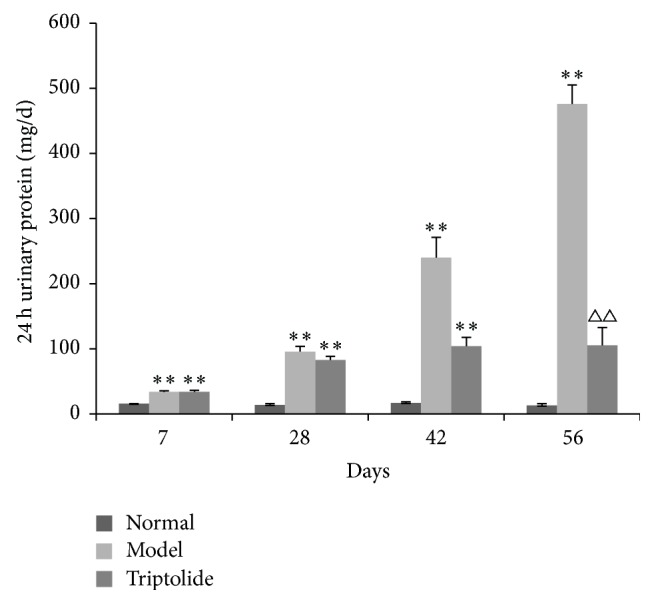
Triptolide treatment attenuated 24 h urinary protein excretion in rats with adriamycin-induced nephropathy. (Compared to the normal group: ^*∗*^ *P* < 0.05, ^*∗∗*^ *P* < 0.01; compared to the model group: ^△△^ *P* < 0.01.)

Urinary protein excretion of the normal group remained at a relatively low level. At 1 week after the nephropathic symptoms appeared, urinary protein levels increased significantly in the model and triptolide groups and became significantly higher (*P* < 0.05) than those in the normal group. At 8 weeks after the establishment of nephropathy, urinary protein levels in the triptolide group significantly decreased (*P* < 0.01), compared to those in the model group (Figure 1). These results show that triptolide could decrease urinary protein excretion in rats with adriamycin-induced nephropathy.

### 3.2. Triptolide Improved Renal Function without Apparent Adverse Effects on the Liver

**Figure 2:**
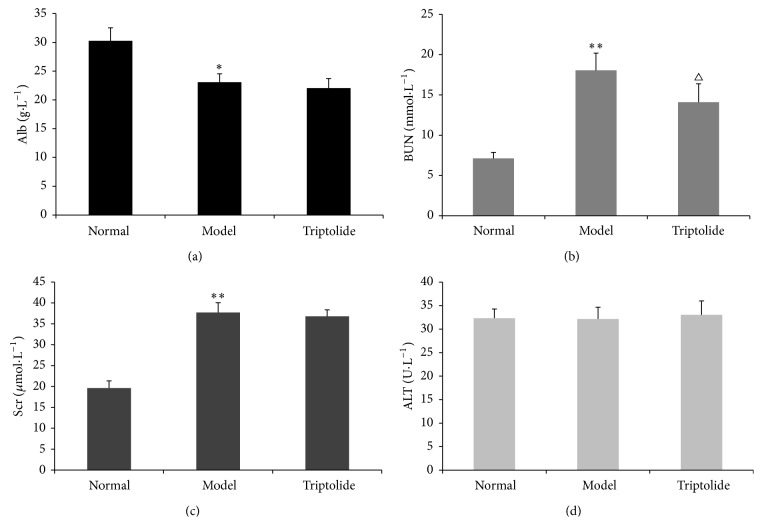
Triptolide improved the renal function of rats with adriamycin-induced nephropathy without apparent adverse effects on the liver. Alb (a), BUN (b), Scr (c), and ALT (d) in normal, model, and triptolide groups. (Compared to the normal group: ^*∗*^ *P* < 0.05, ^*∗∗*^ *P* < 0.01; compared to the model group: ^△^ *P* < 0.05.)

Compared to the normal group, Alb levels in the model group were decreased (*P* < 0.05), while BUN and Scr levels were significantly increased (*P* < 0.01). Compared with the model group, BUN levels in the triptolide group were decreased, and the difference was statistically significant (*P* < 0.05); however, no obvious differences in levels of Scr were observed in these groups. There were no statistically significant differences in ALT among the three groups (Figure 2). These results showed that triptolide could improve the renal function of rats with adriamycin-induced nephropathy without apparent adverse effects on the liver.

### 3.3. Triptolide Ameliorated Renal Histopathology in Rats with Adriamycin-Induced Nephropathy

**Figure 3:**
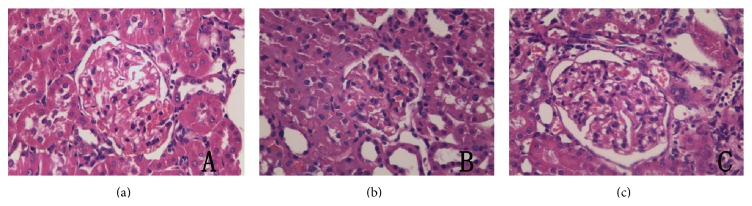
Triptolide ameliorated renal histopathological changes in rats with adriamycin-induced nephropathy. (a) Normal glomerular morphology in a rat from the normal group (here and in the remaining panels of this figure: HE, magnification ×400). (b) Glomerular morphology in a rat from the model group. (c) Glomerular morphology in a rat from the triptolide group.

As determined by light microscopy, glomerular morphology in the normal group appeared normal. However, in the model group, we observed hypertrophic segments of the glomerulus, balloon adhesions, capillary lumen stenosis, and mild-moderate interstitial fibrosis. In the triptolide group, glomerular capillary loops opened well and only a mild hyperplasia of mesangial cells was seen in parts of the glomerulus (Figure 3).

### 3.4. Triptolide Ameliorated Podocyte Foot Process Effacement in Rats with Adriamycin-Induced Nephropathy

**Figure 4:**
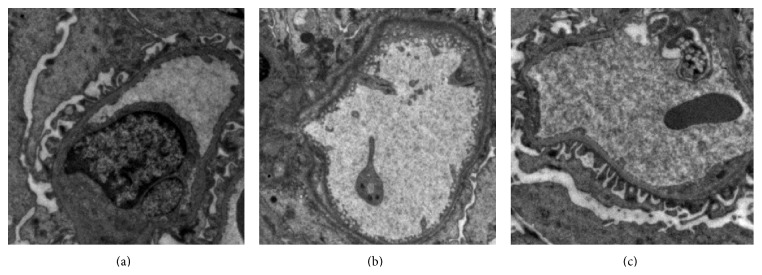
Triptolide ameliorated podocyte foot process effacement in rats with adriamycin-induced nephropathy. (a) No significant abnormalities were detected in podocytes of a rat from the normal group (here and in the remaining panels of this figure: magnification ×15,000). (b) Vacuolated podocytes and extensive podocyte foot process fusion observed in a rat from the model group. (c) Apparent absence of abnormal podocytes in a rat from the triptolide group.

In the normal group, glomerular podocytes showed no significant abnormalities, whereas in the model group podocytes appeared vacuolated with extensive podocyte foot process fusion. In the triptolide group, no obviously abnormal podocytes were observed. These findings indicated the presence of a significant podocyte injury in rats with adriamycin nephropathy and that triptolide could potently alleviate this damage (Figure 4).

### 3.5. Triptolide Upregulated the Absorbance Integral of Nephrin in the Kidney Cortex

**Figure 5:**
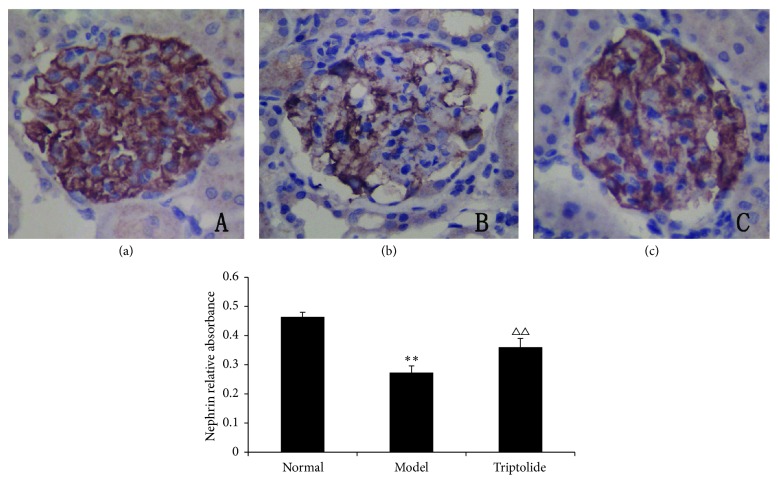
Triptolide upregulated the expression of nephrin in the kidney cortex. (Compared to the normal group: ^*∗∗*^ *P* < 0.01; compared to the model group: ^△△^ *P* < 0.01.)

Nephrin was abundantly expressed in glomerular podocytes in each group. The stained area for nephrin in glomeruli of model rats was significantly reduced and lighter in color. The staining was darker in the triptolide group than in the model group. At the same time, when compared to the normal group, the staining of the triptolide group was less intense, and the area stained was smaller. Semiquantitative immunohistochemistry analysis revealed significant differences between the normal and model groups (*P* < 0.01) and similar differences existed between the model group and triptolide group (*P* < 0.01). These results indicated that triptolide upregulated the expression of nephrin in the kidney cortex (Figure 5).

### 3.6. Triptolide Upregulated Expression of Nephrin in the Kidney Cortex

**Figure 6:**
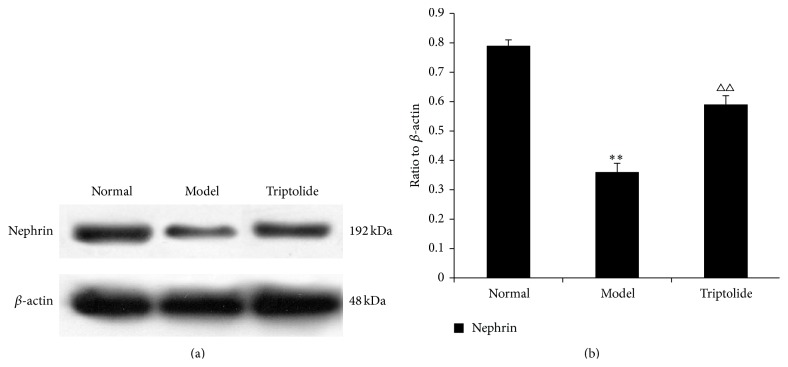
Triptolide upregulated the expression of nephrin. The level of the nephrin protein is expressed in relation to that of *β*-actin. (a) The data show representative blots of nephrin and *β*-actin from three independent experiments. (b) Graphs illustrate nephrin/*β*-actin ratios. Data are expressed as the mean ± S.E.M. (Compared to the normal group: ^*∗∗*^ *P* < 0.01; compared to the model group: ^△△^ *P* < 0.01.)

Compared to that observed in the normal group, expression of nephrin in renal tissues of rats with adriamycin-induced nephropathy was downregulated (*P* < 0.01). However, triptolide potently reversed this deficit by upregulating nephrin expression (*P* < 0.01) (Figure 6).

### 3.7. Renal Cortex miRNA Chip Analysis of Each Group

**Figure 7:**
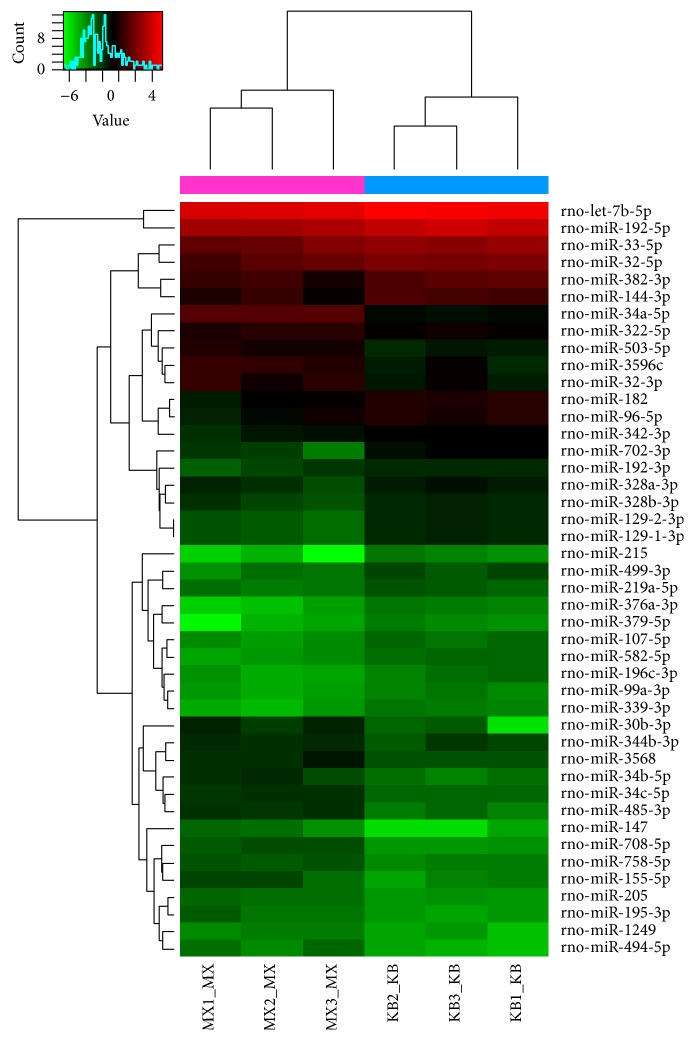
Hierarchical clustering of the normal and model groups. Differential miRNA expression profiles were observed. Red indicates upregulated miRNAs (higher expression) and green indicates downregulated miRNAs (lower expression); the aggregation tree is above the sample and the miRNA aggregation tree is on the left. MX: the model group; KB: the normal group.

**Figure 8:**
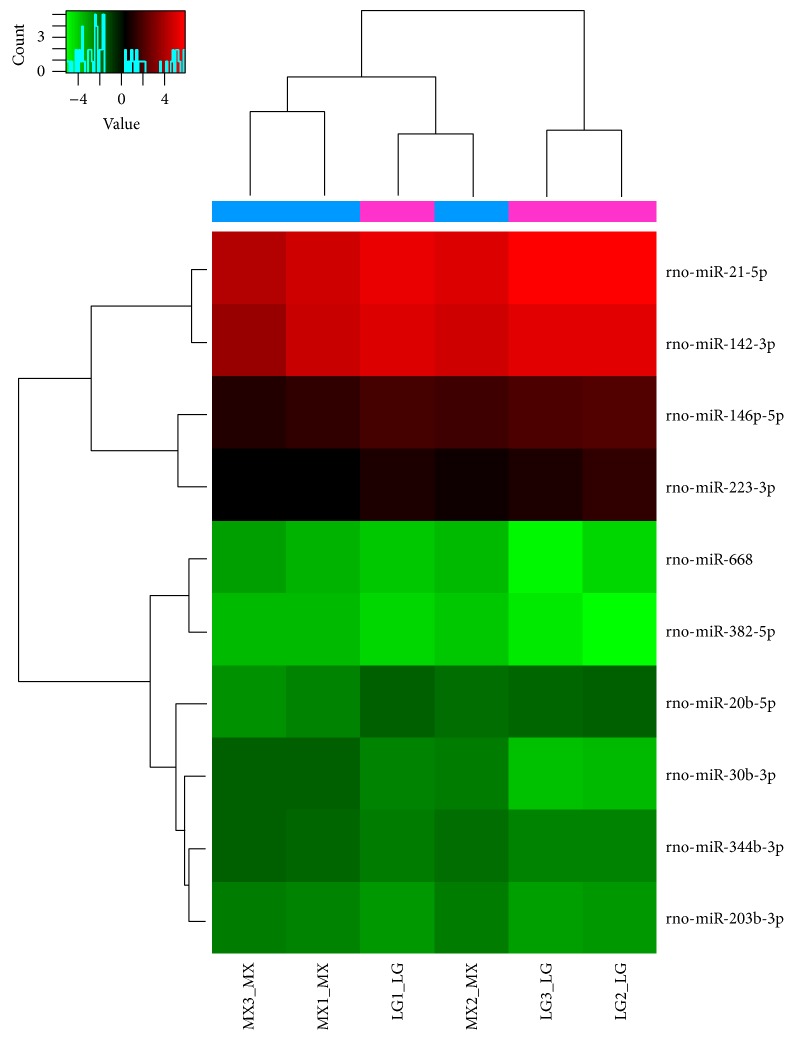
Hierarchical clustering of the model and triptolide groups. Differential miRNA expression profiles were observed. MX: the model group; LG: the triptolide group.

Differential expression of miRNAs was seen in renal cortex samples from various groups. The original value of each sample chip was scanned against the background, normalized, and the latter value was used in further analysis. Values in the normal, model, and triptolide groups were compared to each other. Samples between two groups that passed fold-change filtering (fold change of >1.5) and with *P* values < 0.05 were marked as significantly different miRNAs. Chip analysis showed that, compared to that observed in the normal group, 19 miRNAs were significantly upregulated (rno-miR-344b-3p, rno-miR-195-3p, rno-miR-30b-3p, rno-miR-34a-5p, etc.) and 24 miRNAs were significantly downregulated (rno-miR-192-3p, rno-miR-192-5p, rno-miR-33-5p, rno-miR-196c-3p, etc.) in the model group (Tables 1 and 2, and Figure 7). Treatment with triptolide enhanced expression of five miRNAs (rno-miR-146b-5p, rno-miR-20b-5p, rno-miR-142-3p, rno-miR-223-3p, and rno-miR-21-5p), while that of five other miRNAs (rno-miR-668, rno-miR-203-3p, rno-miR-382-5p, rno-miR-344b-3p, and rno-miR-30b-3p) was significantly downregulated (Tables 3 and 4, and Figure 8). Among them, rno-miR-344b-3p and rno-miR-30b-3p were highly expressed in the model group but poorly expressed in the triptolide group. Clustering figures illustrate the results of the layer cluster analysis of the relationships between samples and the miRNAs. Each line represents one miRNA molecule and each column represents a sample. Red indicates an upregulated miRNA (higher expression) and green represents a downregulated miRNA (lower expression). There is an aggregation tree above the sample, while the miRNA aggregation tree is on the left. These results indicated the expression of rno-miR-344b-3p, rno-miR-195-3p, rno-miR-30b-3p, and rno-miR-34a-5p was significantly upregulated in rats with adriamycin-induced nephropathy, whereas triptolide treatment could reverse the elevated expression of rno-miR-344b-3p and rno-miR-30b-3p to normal levels.

### 3.8. Validation of Microarray Results by Reverse Transcription PCR

**Figure 9:**
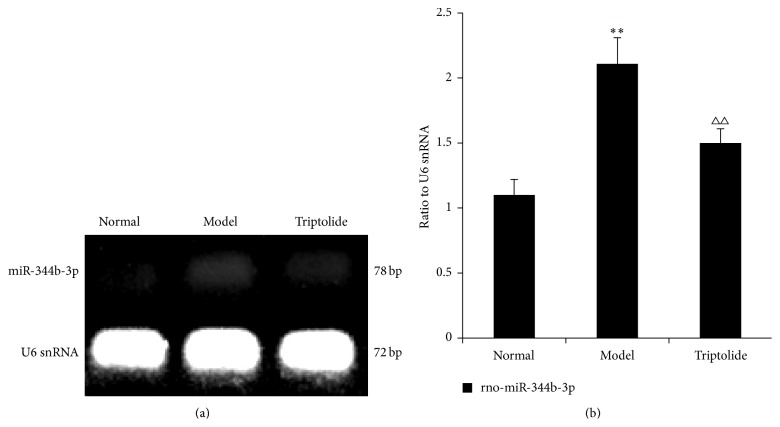
Differential expression of rno-miR-344b-3p in each group. The level of rno-miR-344b-3p expression is expressed in relation to that of U6 snRNA. (a) The data show representative agarose gel electrophoretic patterns from one of the three experiments. (b) Graphs illustrate rno-miR-344b-3p/U6 snRNA ratios. Data are expressed as the mean ± S.E.M. (Compared to the normal group: ^*∗∗*^ *P* < 0.01; compared to the model group, ^△△^ *P* < 0.01.)

**Figure 10:**
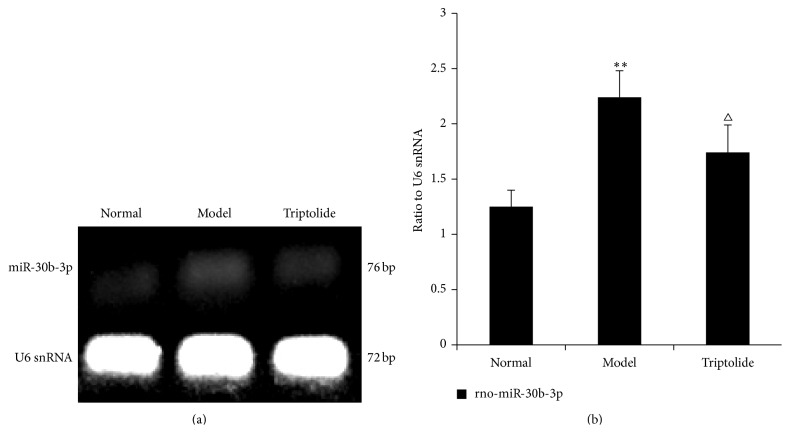
Differential expression of rno-miR-30b-3p in each group. The level of rno-miR-30b-3p expression is expressed in relation to that of U6 snRNA. (a) The data show representative agarose gel electrophoretic patterns from one of three experiments. (b) Graphs illustrate rno-miR-30b-3p/U6 snRNA ratios. Data are expressed as the mean ± S.E.M. (Compared to the normal group: ^*∗∗*^ *P* < 0.01; compared to the model group, ^△^ *P* < 0.05.)

RT-PCR was used to confirm differential expression of miRNAs in each group. Rno-miR-344b-3p and rno-miR-30b-3p were selected as the most differentially expressed miRNAs. We found that expression of rno-miR-344b-3p and rno-miR-30b-3p in the model group was upregulated compared to that observed in the normal group (*P* < 0.01). This increase in expression of these two miRNAs was reversed by triptolide treatment (*P* < 0.01 or *P* < 0.05) (Figures 9 and 10). These findings indicated that the results of RT-PCR detection were consistent with those of the analysis from miRNA chip experiments.

## 4. Discussion

Adriamycin-induced nephropathy in rats is a classic model for kidney pathology. In the early stage, substantial amount of proteinuria and podocyte injury is observed. At later stages, glomerular fibrosis, glomerulosclerosis, and renal failure are seen [25]. In our study, massive proteinuria, pathological damage in renal tissue, podocyte foot process effacement, and downregulation of nephrin were observed in rats with adriamycin-induced nephropathy. These results were consistent with those of previously published reports [26, 27]. In earlier studies, liver toxicity, nephrotoxicity, and infertility were considered the major side effects of the pharmacological constituents of* Tripterygium wilfordii Hook F *[28, 29]. In our study, we saw no apparent adverse effects on the liver; moreover, triptolide improved renal function to some extent. It is possible that previously observed side effects were due to higher doses of triptolide and the selection of different animal models.

A recent study [30] suggests that podocyte injury is one of the factors initiating glomerular sclerosis. The slit membrane is the outermost layer of the glomerular filtration barrier and its protein composition is crucial for the ability of foot processes to wrap around capillaries and carry out barrier function. If the slit membrane protein expression is abnormal, the normal structure of foot processes changes and the integrity of the glomerular filtration membrane is lost. Collectively, these phenomena eventually lead to proteinuria.

Therefore, normal expression of the podocyte protein nephrin is important to control proteinuria [31]. Kuusniemi et al. [32] found that mutations in the* NPSH1* gene could lead to the abnormal expression of nephrin, thereby causing podocyte detachment and, subsequently, proteinuria. Zhang et al. [33] revealed that upregulation of podocyte nephrin expression could reduce urinary protein levels in diabetic rats. Mao et al. [34] showed that glomerular expression of nephrin and CD2AP plays an important part in the pathogenesis of IgA nephropathy in Chinese children. Therefore, there is substantial evidence supporting the existence of a direct relationship between the abnormal expression of nephrin and the loss of podocyte function.

miRNAs are a cluster of small noncoding RNA molecules that play an important role in a variety of physiological and pathological processes in humans [35–37]. Recent studies showed that miRNAs could regulate renal development [38–40] and play an important role in maintaining and adjusting the stability of the structure and function of the kidney.

Biochip technology is uniquely amenable to high-throughput screening, data integration, and miniaturization. Recently it has been successfully applied for the detection of cell or tissue miRNA expression. In the present study, we sought to determine kidney-specific miRNA and use the data to monitor the development of adriamycin-induced nephropathy in an experimental rat model that has gained widespread international recognition. We initially analyzed miRNA expression profiles in the three experimental rat groups (control, adriamycin-induced nephropathy model, and triptolide-treated model) using miRNA chips and subsequently verified the observed differential miRNA profiles by RT-PCR. The aim of our study was to determine expression of relevant miRNAs in rats with adriamycin-induced nephropathy, which could provide the basis for further study of their protective function in podocytes. Interpretation of microarray data may be hampered by false positive results, experimental deviations because of sample mixing, and other reasons. Therefore, having revealed differential miRNA expression profiles using microarray chips, we confirmed these differences by analyzing the expression of selected miRNAs in renal tissues from the normal, model, and triptolide groups by RT-PCR. We observed that expression of rno-miR-344b-3p and rno-miR-30b-3p in renal tissues from the model group rats was enhanced compared to that observed in the control group. Furthermore, we demonstrated that treatment with triptolide could reverse increased expression of these two miRNAs in mice with adriamycin-induced nephropathy. These findings were consistent with the results of earlier screening with microarray chips; hence, we concluded that microarray analysis of differentially expressed miRNAs was accurate and reliable.

It has been shown that miRNAs participate in diverse biological and pathological processes. It is known that dysregulation of miRNAs is associated with kidney disease. For example, it has been shown that miR-21 expression is downregulated in early diabetic nephropathy [41], while miRNA-15a is closely associated with polycystic kidney disease [42]. miR-141, miR-200a, miR-192, and miR-205 are upregulated in patients with hypertensive glomerulosclerosis [43]. Members of the miRNA-30 family are highly expressed in the kidneys of humans and rats [44] and changes in their expression may result in glomerular disease [45]. Luo et al. [46] reported that miRNA-30a was expressed at significantly higher levels in the serum and urine of primary nephrotic syndrome children; however, its expression decreased after hormone therapy and proteinuria remission. miRNA-344 has not been reported to be associated with renal disease, although miRNA-344 can activate Wnt/*β*-catenin signaling and inhibit adipocyte differentiation [47].

Our experiments showed that treatment with triptolide could significantly improve proteinuria in rats with adriamycin-induced nephropathy. The apparent mechanism of podocyte protection is associated with the upregulation of nephrin protein expression in renal tissue. miRNA chip analysis suggested that expression of rno-miR-344b-3p and rno-miR-30b-3p is enhanced in the renal tissue of rats with adriamycin-induced nephropathy and that triptolide treatment can reverse this upregulation in the expression of rno-miR-344b-3p and rno-miR-30b-3p. Furthermore, we confirmed the differential expression of these miRNAs using RT-PCR. We had reasons to believe that expression of rno-miR-344b-3p and rno-miR-30b-3p may play an important role in the protective action of triptolide toward podocytes. Therefore, we speculated that the pathological mechanism of proteinuria might be related to the abnormal expression of miRNAs, which would cause downregulated expression of podocyte nephrin, thereby affecting podocyte function. Triptolide treatment may have upregulated the expression of podocyte nephrin by regulating relevant miRNAs. Upregulation of nephrin, in turn, helped maintain the normal structure of the glomerular filtration membrane and played a role in protecting podocytes.

## 5. Conclusion

In conclusion, triptolide significantly attenuated podocyte injury in rats with adriamycin-induced nephropathy by regulating the expression of miRNA-344-3p and miRNA-30b-3p. It demonstrates that miRNA-344-3p and miRNA-30b-3p might be potential therapeutic targets in the treatment of CKD.

## Figures and Tables

**Table 1:** Upregulated miRNAs in the model group compared with the normal group.

ID	Name	Fold change	*P* value	FDR
46917	rno-miR-205	1.92457	0.00000	0.00459
11074	rno-miR-34c-5p	2.29828	0.00029	0.07672
148480	rno-miR-494-5p	2.76551	0.01961	0.27645
148099	rno-miR-344b-3p	1.52253	0.03831	0.29845
46210	rno-miR-1249	1.91770	0.01733	0.26785
29190	rno-miR-708-5p	3.01526	0.00164	0.14709
29153	rno-miR-34b-5p	2.78153	0.01647	0.26294
42723	rno-miR-195-3p	2.19307	0.01701	0.26686
14288	rno-miR-503-5p	2.43302	0.00351	0.17969
29575	rno-miR-32-3p	2.21251	0.04169	0.30459
168586	rno-miR-34a-5p	4.43281	0.00000	0.00248
42694	rno-miR-485-3p	3.08079	0.00089	0.13137
148343	rno-miR-3568	1.94418	0.03106	0.29127
27558	rno-miR-155-5p	2.53069	0.03852	0.29845
168968	rno-miR-147	3.65262	0.02648	0.28483
13150	rno-miR-322-5p	1.54620	0.00811	0.23523
42626	rno-miR-30b-3p	3.18227	0.01956	0.27645
148139	rno-miR-3596c	2.77024	0.00653	0.20639
148068	rno-miR-758-5p	1.92262	0.00069	0.12406

ID: array ID of the probes; each miRNA always has its unique probe; name: the name of each miRNA; fold change: the ratio of normalized intensities between two conditions (use normalized data, ratio scale); *P* value: *t*-test result between samples in different groups; FDR: FDR is calculated from Benjamini Hochberg FDR. Fold change cutoff: 1.5; *P* value cutoff: 0.05.

**Table 2:** Downregulated miRNAs in the model group compared with the normal group.

ID	Name	Fold change	*P* value	FDR
147165	rno-let-7b-5p	0.62932	0.02113	0.27778
148600	rno-miR-196c-3p	0.47815	0.01955	0.27645
42511	rno-miR-99a-3p	0.61647	0.03477	0.29547
169132	rno-miR-382-3p	0.52745	0.03227	0.29127
148271	rno-miR-328b-3p	0.64070	0.04087	0.30171
145859	rno-miR-33-5p	0.57050	0.02875	0.28933
10975	rno-miR-182	0.52694	0.01030	0.23815
29802	rno-miR-144-3p	0.57609	0.03511	0.29547
17732	rno-miR-192-5p	0.57920	0.00873	0.23814
14303	rno-miR-376a-3p	0.37616	0.00497	0.18890
32884	rno-miR-342-3p	0.64155	0.01882	0.27645
42509	rno-miR-219a-5p	0.63313	0.02686	0.28535
11093	rno-miR-379-5p	0.44332	0.03601	0.29547
11053	rno-miR-32-5p	0.57241	0.02593	0.28172
42912	rno-miR-339-3p	0.50919	0.00519	0.18890
145692	rno-miR-499-3p	0.46762	0.01023	0.23815
147536	rno-miR-107-5p	0.56484	0.00326	0.17934
148069	rno-miR-129-1-3p/ rno-miR-129-2-3p	0.42185	0.00163	0.14709
148588	rno-miR-192-3p	0.62624	0.03383	0.29353
13147	rno-miR-96-5p	0.53033	0.03272	0.29139
11210	rno-miR-215	0.29641	0.01020	0.23815
42800	rno-miR-582-5p	0.50143	0.00517	0.18890
145640	rno-miR-328a-3p	0.60422	0.04310	0.30953
148011	rno-miR-702-3p	0.32394	0.00381	0.18767

**Table 3:** Upregulated miRNAs in the triptolide group compared with the model group.

ID	Name	Fold change	*P* value	FDR
10306	rno-miR-146b-5p	1.50393	0.03006	0.95342
42640	rno-miR-20b-5p	1.55392	0.02002	0.95342
10947	rno-miR-142-3p	1.65281	0.04224	0.95342
11024	rno-miR-223-3p	1.58816	0.03019	0.95342
147506	rno-miR-21-5p	1.95299	0.02303	0.95342

**Table 4:** Downregulated miRNAs in the triptolide group compared with the model group.

ID	Name	Fold change	*P* value	FDR
145701	rno-miR-668	0.51649	0.04825	0.95342
148012	rno-miR-203b-3p	0.65810	0.00558	0.95342
145643	rno-miR-382-5p	0.53341	0.01850	0.95342
148099	rno-miR-344b-3p	0.65787	0.00652	0.95342
42626	rno-miR-30b-3p	0.39387	0.02813	0.95342
